# Cancer vaccines: platforms and current progress

**DOI:** 10.1186/s43556-024-00241-8

**Published:** 2025-01-10

**Authors:** Wanting Lei, Kexun Zhou, Ye Lei, Qiu Li, Hong Zhu

**Affiliations:** 1 Department of Medical Oncology, Cancer Center, West China Hospital, Sichuan University, Chengdu, 610041 Sichuan China; 2 College of Liberal Arts, Neijiang Normal University, Neijiang, 641100 Sichuan China; 3 Division of Abdominal Tumor Multimodality Treatment, Cancer Center, West China Hospital, Sichuan University, Chengdu, 610041 Sichuan China

**Keywords:** Cancer vaccine, MRNA vaccines, Tumor antigens, Neoantigens, Adjuvants, Tumor microenvironment, Immune memory, Combined therapies, Adverse events

## Abstract

Cancer vaccines, crucial in the immunotherapeutic landscape, are bifurcated into preventive and therapeutic types, both integral to combating oncogenesis. Preventive cancer vaccines, like those against HPV and HBV, reduce the incidence of virus-associated cancers, while therapeutic cancer vaccines aim to activate dendritic cells and cytotoxic T lymphocytes for durable anti-tumor immunity. Recent advancements in vaccine platforms, such as synthetic peptides, mRNA, DNA, cellular, and nano-vaccines, have enhanced antigen presentation and immune activation. Despite the US Food and Drug Administration approval for several vaccines, the full therapeutic potential remains unrealized due to challenges such as antigen selection, tumor-mediated immunosuppression, and optimization of delivery systems. This review provides a comprehensive analysis of the aims and implications of preventive and therapeutic cancer vaccine, the innovative discovery of neoantigens enhancing vaccine specificity, and the latest strides in vaccine delivery platforms. It also critically evaluates the role of adjuvants in enhancing immunogenicity and mitigating the immunosuppressive tumor microenvironment. The review further examines the synergistic potential of combining cancer vaccines with other therapies, such as chemotherapy, radiotherapy, and immune checkpoint inhibitors, to improve therapeutic outcomes. Overcoming barriers such as effective antigen identification, immunosuppressive microenvironments, and adverse effects is critical for advancing vaccine development. By addressing these challenges, cancer vaccines can offer significant improvements in patient outcomes and broaden the scope of personalized cancer immunotherapy.

## Background

**Fig. 1:**
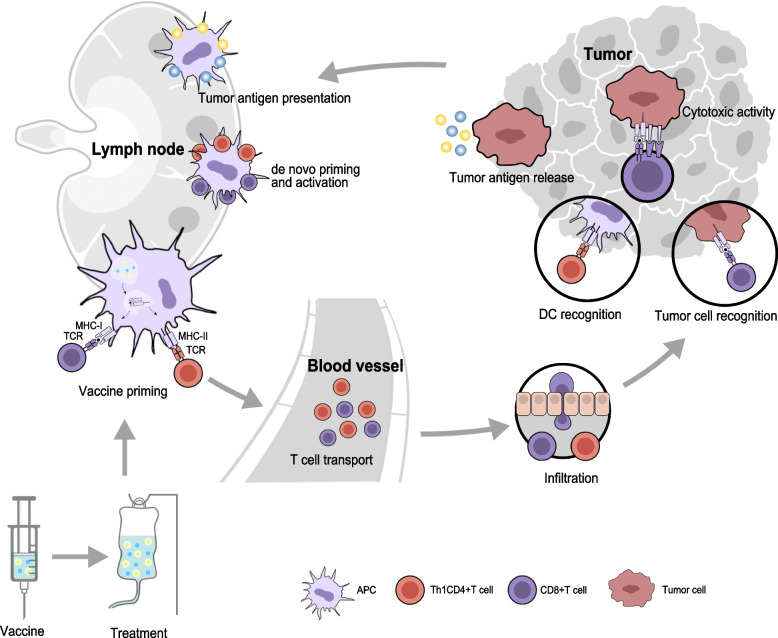
Immune response induced by therapeutic cancer vaccines. The normal immune system of the body is able to kill tumor cells by activating T cells to recognise the surface antigens of tumor cells and undergo antigen–antibody reactions. Therapeutic cancer vaccines are to further enhance this effect by introducing the tumor antigens into the patient's body in various forms, such as patient-derived tumor cells, tumor-associated proteins or peptides, and genes expressing tumor antigens, etc., which are prepared as cancer vaccine preparations by synthetically supplementing them with adjuvants or by using DCs as a delivery vehicle. After the vaccine enters the body through the blood circulation, tumor antigens targeted by the vaccine are efficiently processed by specialized APCs, such as DCs. The MHC of APCs presents antigens on their surface, and the MHC complex activates antigen-specific T cells by binding to the T cell receptor (TCR) on the surface of the T cell, which activate CTL and CD4 + Th1 cells, thereby safely, persistently and specifically destroying tumor cells and inhibiting tumor growth. APC, antigen presenting cell; DC, dendritic cell

Cancer vaccines, categorized as preventive or therapeutic, represent a pivotal immunotherapeutic strategy aimed at enhancing anti-tumor immunity. Preventive cancer vaccines, such as those targeting human papillomavirus (HPV) and hepatitis B virus (HBV), are designed to reduce cancer incidence by preventing virus-induced malignancies [1]. Therapeutic cancer vaccines, in contrast, activate dendritic cells (DCs) and promote infiltration of cytotoxic T lymphocytes (CTLs) to elicit immune responses against tumor-associated antigens (TAAs) and tumor-specific antigens (TSAs) (Fig. 1). These vaccines further contribute to long-term immune memory, providing a robust defense against tumor recurrence.

The exploration of cancer vaccines originated in the late nineteenth century, when William B. Coley observed tumor regression following treatment with bacterial toxins, marking the first evidence of immune-based cancer therapy [2]. Later, the 1980s marked the advent of the first therapeutic cancer vaccine, the BCG vaccine, proving effective in bladder cancer treatment [3]. Early studies focused on identifying TSAs and trying to use them to activate the body's immune response to attack cancer cells. Following the approval of Sipuleucel-T in 2010, therapeutic vaccine development boomed [4]. However, the failure of several shared-antigen vaccines in large clinical trials turned researchers' attention to more personalized approaches [5]. In recent years, advancements in genetic engineering have revolutionized the field, particularly with mRNA vaccines showing promise against cancers such as glioblastoma and melanoma. The approval of WGc-043, the first mRNA vaccine for Epstein-Barr virus-associated cancers, and the clinical trials of mRNA-4157 in melanoma patients highlight the rapid progress in this domain [6, 7]. These milestones underscore the growing potential of cancer vaccines in improving therapeutic outcomes.

The increasing incidence of cancer worldwide has made the development and application of cancer vaccines an important public health issue. According to the World Health Organization (WHO), cancer is one of the leading causes of death worldwide, and the number of new cancer cases is expected to reach 35 million by 2050 [8]. Cancer vaccine therapy has fewer side effects than traditional chemotherapy and radiation and induces immune memory, providing long-term protection and improving patient survival and quality of life [2, 9]. By targeting a broad range of antigens, cancer vaccines offering the potential to address limitations of existing immunotherapies like immune checkpoint inhibitors (ICIs) and adoptive cell therapies (ACTs) and complement other therapies [10–14]. Advances in vaccine platforms, neoantigen discovery, and combination therapies are enhancing their effectiveness, promising improved outcomes for cancer patients worldwide.

In light of the distinctive advantages of cancer vaccines, this review systematically summarizes their classification, newly identified antigens that may enhance vaccine specificity, recent advancements in delivery platforms, adjuvants that improve efficacy, and promising combination therapies. Additionally, we address the challenges encountered in clinical practice and explore potential future directions, providing insights into the integration of vaccines into cancer therapeutics.

## Preventive and therapeutic vaccines

Vaccines are biological agents derived from diverse sources, including bacteria, viruses, and tumor cells, designed to elicit a specific immune response that aims to either prevent or treat infections from designated pathogens [15].

Preventive vaccines are instrumental in safeguarding healthy individuals against cancers linked to certain viruses, analogous to how the influenza vaccine protects against viral infection. The efficacy of these vaccines is contingent upon their administration prior to viral exposure. Currently, the FDA has sanctioned two vaccines for cancer prevention. The first, the HPV vaccine, reduces the incidence of HPV-associated cancers, such as cervical, anal, and oral cancers, among others [16]. Multiple HPV vaccine formulations exist, catering to different age groups and providing tailored protection. It’s essential to note, however, that HPV vaccination is a supplement, not a substitute, for regular cervical cancer screening. The second FDA-approved cancer vaccine targets the HBV, which is known to cause liver cancer. The hepatitis B vaccine is crucial in preventing HBV infection and its serious sequelae, including liver cancer, offering protection across various demographics. These cancer vaccines exemplify the importance of preventive measures in cancer control strategies, highlighting a shift towards interventions that can prevent rather than treat the disease, thus potentially saving millions of lives globally.

In parallel with vaccines targeting infectious diseases, therapeutic cancer vaccines aim to activate the immune system to recognize and eradicate cancer cells. These malignant cells possess distinct proteins, or antigens, which distinguish them from normal cells. Therapeutic cancer vaccines enhance the immune reaction by utilizing either non-specific pro-inflammatory agents and adjuvants to amplify existing anti-tumor defenses, or by triggering a new immune response targeted at specific tumor-associated antigens [17]. An optimal therapeutic cancer vaccine should encompass several critical attributes. Primarily, it must efficiently deliver high-quality antigens to dendritic cells (DCs), particularly TSAs that exhibit strong binding affinities to human leukocyte antigens (HLAs). Neoantigens with these properties are crucial as they elicit robust immune responses, effectively circumvent tumor immune evasion, and minimize adverse effects on non-target tissues [18, 19]. Furthermore, robust activation of DCs is critical, especially as the tumor microenvironment (TME) often impairs DC functionality due to its suppressive nature. To overcome this, strategic inclusion of adjuvants is necessary to enhance the immunogenic capabilities of DCs. Additionally, these vaccines should continuously stimulate the powerful and sustained action of cytotoxic T lymphocytes (CTLs), which are essential for maintaining long-term antitumor immunity [20, 21].

## Appropriate tumor antigen in cancer vaccine

Principal components of tumor vaccines generally consist of tumor antigens, delivery platforms and immune adjuvants. The effectiveness of cancer vaccines hinges primarily on the precise detection of high-quality tumor antigens, which are abundantly present in specific tumor types or are crucial as they trigger the immune responses essential for the formulation and efficacy of cancer vaccines [17]. These antigens are divided into categories of shared and personalized based on their expression frequency [22]. Commonly, shared antigens are associated with common hotspot mutations within HLA alleles, primarily encompassing TAAs and TSAs [23].

TAAs are usually overexpressed in tumor cells, typically including carcinoembryonic antigen (CEA), prostate-specific antigen (PSA), alpha-fetoprotein (AFP), cancer antigen 125 (CA125), and mucin 1 (MUC1). However, the central immune tolerance mechanism in the thymus may lead to the elimination of T cells recognizing TAAs during their development, potentially limiting the efficacy of cancer vaccine. Furthermore, as TAAs are also present in normal tissues, there is an increased risk of vaccine-induced autoimmune toxicity [24]. As early as 1996, the FDA sanctioned the use of recombinant CEA-pox and DNA vaccines for colorectal cancer (CRC) treatment [25, 26]. Nonetheless, challenges such as limited intensity and duration of action hinder the effectiveness of CEA-based cancer vaccines, which necessitates the development of second-generation alternatives. In a phase I trial, Nakagawa et al. explored an AFP peptide vaccine for advanced hepatocellular carcinoma (HCC), observing an immune response in five patients, one of whom achieved complete remission (CR) and eight others maintained stable disease (SD) without serious AEs [27]. Another study on a bivalent GD2/GD3 vaccine for neuroblastoma showed it was both enduring and effective, presenting a favorable safety profile during a phase II trial [28]. Additionally, BNT111, a liposomal RNA vaccine, targets four non-mutated tumor-associated antigens, including New York esophageal squamous cell carcinoma 1 (NY-ESO-1), tyrosinase, MAGE family member A3 (MAGE-A3), and transmembrane phosphatase with tensin homology (TPTE), in melanoma. This vaccine demonstrated a 16% objective response rate (ORR) as a monotherapy and a 35% ORR in combination with PD-1 inhibitors [29]. Currently, a phase II trial is underway to assess its combination with cemiplimab for patients with anti-PD-1-refractory unresectable stage III/IV melanoma (NCT04526899).

Conversely, TSAs are distinct to cancer cells, enabling effective tumor-specific T-cell immune response and minimizing on-target off-tumor toxicity. Classical TSAs include RAS, p53, epidermal growth factor receptor (EGFR), etc. Rindopepimut (CDX-110), a peptide vaccine targeting the EGFRvIII mutant present in approximately 25–30% of GBMs, showed promising results in a phase II trial when combined with temozolomide (TMZ), significantly improving disease-free survival (DFS) and OS [30]. However, the subsequent phase III trial failed to conquer these findings [31]. The current emphasis in neoantigen cancer vaccine development has shifted to targeting mutated tumor cells with non-germline-encoded TSAs [32]. Techniques such as mass spectrometry (MS) and whole exome sequencing (WES) are crucial for identifying unique epitopes suitable for personalized vaccines. WES and RNA sequencing integration is instrumental for spotting somatic mutations and analyzing mutant allele expression via next-generation sequencing (NGS) [33, 34]. Furthermore, MS significantly boosts the precision in detecting processed immunopeptides and substantially reduces the typical errors found with traditional predictive methods [35]. Continuous optimization of bioinformatics algorithms is also crucial in this field. Algorithms like NetChop, NetCTL, and NetMHCpan are critical for verifying key parameters including peptide shear, translocation, and their binding affinity to MHC-I molecules. These algorithms adeptly predict neoantigens from both synonymous and non-synonymous single-nucleotide variants, including insertions and deletions [4, 36]. Additionally, extensive research developed proprietary AI-based algorithms, aiming to select immunogenic epitopes, thereby promoting the progress of next-generation personalized cancer vaccines [37–39].

Contemporary neoantigen targeting approaches focus on patients with a high burden of mutations. For instance, Tedopi, a novel anti-tumor vaccine, demonstrated a one-year survival rate of 46% in a phase III trial, outperforming the standard chemotherapy regimen by 10% [40]. This vaccine achieved orphan drug designation for HLA-A2-positive NSCLC in the U.S. and is currently under evaluation in HLA-A2-positive pancreatic cancer (NCT03806309). Another example is Neo-MoDC, a personalized monocyte-derived DC vaccine containing neoantigens. was administered to a patient with advanced metastatic gastric cancer along with anti-PD-1 therapy, achieving a complete response lasting 25 months. This highlights the potential of this approach [41]. TG4050 is an individualized cancer vaccine tailored with over 30 personalized neoantigens to effectively activate and expand anti-tumor T cell responses, specifically targeting HPV-negative head and neck cancers. Data from the phase I clinical trial indicated that high-risk patients who received the TG4050 vaccine following their initial treatment experienced no relapses, in contrast to unvaccinated patients, who exhibited three relapse events [42].

Given the limited efficacy of ICIs in patients with low tumor mutational burdens, exploring cancer vaccines that activate host effector T cells alongside ICIs to CTL activity presents a viable research direction. This approach could potentially counteract immunotherapy resistance. Moreover, the efficacy of cancer vaccines is contingent upon the precise identification of mutated tumor antigens. As the majority of cancer mutations are exclusive to each patient, it is imperative to initially procure tumor tissues from the patients and sequence their genomes through NGS or nanotechnology, among other methods, to predict mutant peptides that bind to the patient MHC subunits, accurately identify tumor antigens, and subsequently implement a personalized design, thereby effectively reducing the mutation load and enhancing the recognition and response of the immune system to tumor antigens.

## Cancer vaccine platforms

Besides selecting optimal antigens, the choice of delivery platforms is crucial in cancer vaccine development, as targeting antigens accurately to the intended site presents significant challenges. The function of these platforms in cancer vaccines includes enhancing antigen immunogenicity by efficiently presenting them to immune cells like DC and T cells [43]. These platforms protect antigens from degradation due to enzymatic actions, ensuring their activity and recognition by the immune system [44]. Targeted delivery using engineered platforms such as nanoparticle enhances antigen uptake and immune response specificity. Additionally, these platforms can localize delivery to immune cells, minimizing systemic side effects and improving vaccine safety by modulating the timing and location of antigen release, thus enhancing overall vaccine efficacy [45]. Based on composition and delivery method, cancer vaccines are typically categorized into synthetic peptides, DNA/RNA, cellular vaccines, viral vaccines, and nano-vaccines (Table 1). The following sections elaborate on the above.

**Table 1:** Cancer vaccines with available clinical data

Name	Cancer type	Vaccine format	Ref
Gp100	Melanoma	SSP	[46]
WT1	AML	SSP	[47]
GP2	Breast Cancer	SSP	[48]
HPV16 E6/E7	Cervical Cancer	SLP	[49]
HSPPC-96(Vitespen)	RCC	SLP	[50]
NEO-PV-01	Melanoma, NSCLC, and bladder cancer	SLP	[51]
Tedopi	NSCLC, PDAC	SLP	[40]
PolyPEPI1018	CRC	SLP	[52]
12MP	Melanoma	SLP	[53]
GPC3-Vaccine	HCC	SLP	[54]
ADXS-503	NSCLC	SLP	[55]
Cimavax-EGF	HNSCC, NSCLC	SLP	[56]
MRP3765	HCC	SLP	[57]
INO-5401/9012	GBM, Urothelial Carcinoma	DNA	[58]
mRNA-4157	Melanoma	RNA	[59]
JCXH-211	Solid tumors	RNA	[60]
LK101	Lung cancer	RNA	[61]
GRANITE	NSCLC, Gastrointestinal Solid Tumor, Urothelial Carcinoma	RNA	[62]
BI1361849	NSCLC	RNA	[63]
BNT122	Pancreatic Cancer	RNA	[64]
GVAX	Pancreatic Cancer	Autologous cell	[65]
HS-110	NSCLC	Allogeneic cell	[66]
Sipuleucel-T	Prostate Cancer	DC	[67]
DCVax-L	GBM	DC	[68]
DCVAC/OvCa	Ovarian Cancer	DC	[69]
AV-GBM-1	GBM	DC	[70]
Neo-MASCT	HCC	DC	[71]
Neo DCVac	NSCLC, HCC,	DC	[72]
CCL21-DC	NSCLC	DC	[73]
Ilixadencel	HCC	DC	[74]
Neo-MoDC	Gastrointestinal Solid Tumor	DC	[41]
T-VEC	Melanoma, Squamous Cell Carcinoma	Virus	[75]
VRP-HER2	Breast Cancer	Virus	[76]
VBI-1901	GBM	VLP	[77]
HDDT	Mammary Carcinoma	Nano-vaccine	[78]
dClip-LNP/siRNA	Melanoma	Nano-vaccine	[79]
CS-TEMPO-OVA	Melanoma	Nano-vaccine	[80]

*SSP* synthetic short peptide, *AML* acute myeloid leukemia, *SLP* synthetic long peptide, *RCC* renal cell carcinoma, *NSCLC* non-small-cell lung cancer, *PDAC* pancreatic ductal adenocarcinoma, *CRC* colorectal cancer, *HCC* hepatocellular carcinoma, *GBM* glioblastoma, *HNSCC* head and neck squamous cell carcinoma, *DC* dendritic cell

### Synthetic peptide cancer vaccines

Synthetic peptide cancer vaccines, classified into HLA-restricted short peptides and non-HLA-restricted long peptides, are a significant category of cancer vaccines. Short peptide cancer vaccines, comprising fewer than 15 amino acids and derived from a single antigen, are economical to produce and offer benefits like modifiability, biocompatibility, and reduced immunogenicity, which lowers the likelihood of adverse reactions [81–83]. Nonetheless, challenges persist. They circumvent the requirement for antigen presentation by professional antigen-presenting cells (APCs) and bind effectively to MHC-I molecules. However, their interaction with non-professional APCs fails to provide essential co-stimulation, leading to tolerogenic signaling and T cell dysfunction that diminish vaccine immunogenicity [84–86]. Additionally, short peptides do not trigger CD4 + helper T cells, crucial for cytotoxic T lymphocyte (CTL) activation. To boost the immune response, adjuvants such as poly-ICLC are frequently utilized [87].

Clinical trials have demonstrated the potential of short peptide cancer vaccines. For example, the gp100 peptide cancer vaccine, when administered alongside high-dose IL-2, has effectively stimulated tumor-reactive T cell production in the majority of patients, achieving an ORR of 42% [88]. Additionally, both the Wilms’ tumor gene 1 (WT1) peptide cancer vaccine and the Galinpepimut-S peptide cancer vaccine have yielded promising immune response [89]. The GP2 cancer vaccine, targeting HER2/neu and designed for breast cancer, exhibited a notable trend toward improved DFS among HER2-positive patients post-standard treatment. Remarkably, no recurrences were observed in these patients compared to an 89.4% recurrence rate in patients treated with GM-CSF alone, and without significant adverse events reported [46].

Conversely, synthetic long peptide (SLP) cancer vaccines encompass multiple MHC-I binding epitopes that activate numerous T cells simultaneously, eliciting a more robust T cell response with lower toxicity [47]. Specifically, long peptide cancer vaccines targeting HPV16 E6 and E7 sequences have demonstrated minimal toxicity during clinical evaluations [48]. SLP cancer vaccines can also enhance immunogenicity through self-assembled structures, which can form diverse nanostructures that are endocytosed and presented by DCs, thereby stimulating both cellular and humoral immunity [90]. Nevertheless, synthesizing SLP cancer vaccines is complex, with challenges like limited tissue permeability, susceptibility to enzymatic degradation, and rapid clearance, requiring adaptations such as lipid modifications [49, 50, 91].

Recent innovations in peptide cancer vaccines have introduced multivalent, peptide cocktail, and hybrid formats, advancing immune responses by broadening the range and diversity of antigenic epitopes [92]. Vitespen, an autologous tumor-derived heat-shock protein (glycoprotein 96)-peptide complex, emerged as the first cancer vaccine to show a survival benefit in patients with advanced melanoma (M1a and M1b substages) [93]. And early-stage patients with locally advanced renal cell carcinoma (RCC) could also benefit from Vitespen, with improved recurrence-free survival (RFS) [94]. Based on these, Vitespen received approval from the Russian Ministry of Health for patients with RCC at moderate risk of recurrence. Results from the phase Ib clinical trial indicated that NEO-PV-01, containing up to 20 unique neoantigens in synthetic long peptide form, in combination with a PD-1 antibody significantly prolonged survival in patients with advanced melanoma, non-small cell lung cancer (NSCLC), and bladder cancer, compared tonivolumab monotherapy [51]. PolyPEPI1018 cancer vaccine, an SLP vaccine containing twelve unique epitopes derived from seven conserved cancer antigens frequently expressed in metastatic colorectal cancer (mCRC), also elicited durable response against CRC [52]. Furthermore, a phase II clinical trial of novel SLP vaccines for melanoma (NCT00118274) demonstrated a significant enhancement in overall survival (OS) when administered alongside the adjuvant 12MP (targeting CD8 + T cells) and 6MHP (targeting CD4 + T cells) in patients with stage IIB to IV melanoma, with long-term DFS seen in patients with partially recurrent resectable disease [53].

### Nucleic acid cancer vaccines

Nucleic acid cancer vaccines, capable of directly generating the target antigen, elicit a robust CD8 + T-cell response mediated by MHC-I molecules through the transfection of RNA or DNA into host cells [95]. These cancer vaccines are typically divided into DNA and mRNA categories. DNA cancer vaccines, which encode antigens that persist longer in the body, trigger enduring immune response and possess advantages such as greater stability [96, 97]. However, they exhibit lower immunogenicity, and viral vectors (e.g., adenoviruses) are often utilized to enhance DNA transfection efficiency into the nucleus. This reliance on delivery vectors raises concerns regarding potential safety issues, such as vector-mediated genotoxicity, as well as the risk of genetic mutations [96, 98]. INO-5401 is an example of a synthetic DNA cancer vaccine encoding a variety of cancer antigens such as human telomerase reverse transcriptase (hTERT), WT1, and prostate-specific membrane antigen (PSMA). Research by Reardon et al. into INO-5401 and INO-9012 (a synthetic DNA plasmid encoding interleukin-12 [IL-12]) in patients with glioblastomas showed that this approach could trigger a strong immune response that correlates with enhanced survival outcomes [58].

In contrast, mRNA cancer vaccines, which synthesize proteins within the cytoplasm, bypass the need for nuclear entry, thus avoiding some safety concerns linked to DNA cancer vaccines. Typically, these cancer vaccines contain genes for nonstructural proteins that encode viral replicative enzymes, facilitating high levels of RNA amplification and expression in the cytoplasm [99]. The integration of modified ribonucleotides into mRNA helps diminish its innate immunogenicity and increase its stability, attributes beneficial to mRNA cancer vaccines [100, 101]. However, the transient presence of mRNA in the cytoplasm prior to degradation necessitates effective delivery methods like lipid/protamine/mRNA nanoparticles [102, 103]. This underlines the importance of optimizing sequence designs in mRNA-based treatments to mitigate off-target effects [104].

mRNA cancer vaccines have drawn considerable interest in clinical applications. For instance, mRNA-4157/V940, a personalized mRNA cancer vaccine, is currently being evaluated in combination with pembrolizumab for patients with stage III/IV melanoma following complete surgical resection. Recent data indicated that this combination significantly reduces the risk of recurrence or death by 44%, compared to pembrolizumab alone, while maintaining a safety profile consistent with prior phase I trial [59, 105]. JCXH-211, a neoantigenic mRNA cancer vaccine, facilitates the in *vivo* expression of IL-12 at low doses over extended periods, effectively targeting tumor cells, eradicating distal tumors, and mitigating the risk of tumor recurrence. Following the promising outcomes of a phase I clinical trial (NCT05727839), the vaccine has received Investigational New Drug (IND) approval from the FDA [60].

### Cellular cancer vaccines

Cellular cancer vaccines, categorized into autologous and allogeneic categories, form a cornerstone of immunotherapy. Originating from either entire cells or cellular fragments, these cancer vaccines generate a broad immune response by targeting an array of tumor-associated antigens (TAAs) [106]. However, their inherent lack of specificity may diminish the effectiveness of immune response, often necessitating the addition of immunological adjuvants [107]. The GVAX cancer vaccine comprises genetically modified whole tumor cells designed to secrete GM-CSF, a potent immunostimulatory cytokine that enhances DC activation [65]. Despite demonstrating tumor regression and extended survival in preclinical studies, GVAX showed limited efficacy in clinical practice [108–110]. The GVAX-PCa variant, which includes a combination of irradiated PC-3 and LNCaP prostate cancer cell lines overexpressing GM-CSF, has progressed into clinical trials for prostate cancer [111]. A phase I/II study indicated that it stimulated an antigen-specific immune response and was generally well-tolerated [112]. Nevertheless, subsequent phase III trial were halted due to severe AEs and insufficient efficacy [113]. Despite these setbacks, research on GVAX continues. In 2017, a study suggested that combining GVAX with cyclophosphamide and degarelix as neoadjuvant therapy for high-risk prostate cancer could potentially enhance outcomes concerning time-to-prostate-specific antigen (PSA) relapse [114].

Conversely, allogeneic tumor cell cancer vaccines utilize established malignant cell lines, providing a cost-effective and plentiful source of tumor antigens [115, 116]. HS-110, derived from a human lung cancer cell line, bolsters antigen presentation and DC activation. During the DURGA trial, HS-110 in combination with nivolumab achieved a 15% ORR and a 55% disease control rate (DCR) in patients resistant to prior immunotherapies, maintaining a safety profile consistent with nivolumab alone [66]. However, it is worth noting that in the growing field of allogeneic cellular cancer vaccines, the promising results of most phase II trials were not conquered by further phase III trials. This inconsistency underscores a significant gap in translating research findings into clinical application, warranting further examination [117, 118].

DC cancer vaccines, recognized for their robust antigen-presenting capabilities and strong immunogenicity, constitute a vital subset of cellular cancer vaccines. Personalized DC-based neoantigenic cancer vaccines have recently demonstrated substantial anti-tumor efficacy in clinical settings [119–121]. For instance, sipuleucel-T, approved by the FDA in 2010 for metastatic castration-resistant prostate cancer (mCRPC), is generated from patient-derived leukocytes activated in vitro with mesenchymal DCs and a GM-CSF-linked prostatic acid phosphatase (PAP) antigen. In the phase III IMPACT trial involving 512 mCRPC patients, sipuleucel-T demonstrated a median OS of 25.8 months versus 21.7 months in the placebo group [67]. DCVax-L represents another autologous tumor lysate-loaded DC vaccine, notable for its capacity to target a comprehensive antigenic library through tumor lysates, thus optimizing antigen presentation [68, 122, 123]. It showed promising efficacy in a phase III trial involving patients with newly diagnosed gliomas (nGBM) and recurrent gliomas (rGBM). Specifically, the median OS was 19.3 months for the DCVax-L group compared to 16.5 months for the placebo group among nGBM patients, with 5-year survival rates of 13.0% versus 5.7%, respectively. Additionally, patients treated with DCVax-L experienced a 20% relative reduction in mortality risk over time compared to those receiving standard therapy. For rGBM patients, the median OS was 13.2 months for the DCVax-L group, compared to 7.8 months for the placebo group [124]. Another example, LK101, a personalized neoantigen-pulsed DC cancer vaccine, has shown potential benefits as adjuvant therapy in liver cancer, with no grade 1 AEs reported [61]. Currently, a phase Ib/IIa study is underway to assess the safety and efficacy of LK101 in combination with pembrolizumab or durvalumab in advanced non-small cell lung cancer (NSCLC) and small cell lung cancer (SCLC) (NCT05886439), with further results anticipated. These findings underscore the potential of DC vaccines in cancer treatment.

### Viral cancer vaccines

Viruses inherently possess immunogenic properties, and their genetic material can be engineered to carry sequences encoding tumor antigens. Recombinant viral vectors, such as adenoviruses and lysogenic viruses, are particularly effective in transporting these antigens to immune cells, allowing for extensive presentation of tumor antigens and thereby strengthening anti-tumor immune responses [125, 126]. Moreover, these engineered viruses can also rupture tumor cells, releasing antigens and enhancing both the effectiveness of the vaccine and the longevity of immune memory.

Talimogene laherparepvec (T-VEC) is an oncolytic virus engineered to express GM-CSF. The OPTiM study demonstrated that T-VEC significantly improved durable remission rates (DRR) compared to GM-CSF, with a durable remission rate (DRR) of 25.2% in stage III/IV melanoma patients. Additionally, the median OS for T-VEC-treated patients was 41.1 months, compared to 21.5 months for GM-CSF-treated patients [75]. Further analysis indicated that T-VEC not only stimulates systemic immune reactions but also modifies the TME, potentially increasing the effectiveness of subsequent immunotherapies. VRP-HER2, a viral vector-based cancer vaccine encoding HER2, demonstrated promising results in both preclinical and clinical studies. In a breast cancer mouse model, VRP-HER2 vaccination inhibited tumor progression, and further phase I clinical trial corroborated these findings [127]. The prolonged progression-free survival (PFS) was strongly correlated with HER2-specific immune response. Similarly, nadofaragene firadenovec, a non-replicating adenoviral vector cancer vaccine encoding human interferon alpha (IFN-α), held promise for treating non-muscle-invasive bladder cancer, achieving a 53.4% complete remission rate after the first treatment in BCG-naïve patients [128].

Importantly, VBI-1901 employs VBI's enveloped virus-like particles (eVLPs) to specifically target two highly immunogenic cytomegalovirus (CMV) antigens—gB and pp65. Unlike conventional viruses, VLPs lack the viral genome and are incapable of replication, thereby ensuring a superior safety profile. Data derived from a phase I/II study (NCT03382977) indicated that the VBI-1901 cohort achieved a median OS of 12.9 months, in contrast to 8 months in the control group. In light of these compelling clinical trial results, the FDA has conferred fast track designation and orphan drug status upon VBI-1901 for the treatment of recurrent GBM [77]. Based on these impressive results, the FDA has now granted fast track designation and orphan drug status to VBI-1901 for the treatment of recurrent GBM.

### Cancer nano-vaccines

By co-delivering antigens and adjuvants through intelligent nanocarriers, cancer nano-vaccines can facilitate effective targeted delivery, enhance antigen uptake and presentation, and thereby activate specific immune response to eliminate tumor cells [129]. These nano-vaccines are characterized by their nanoscale size, which mirrors that of pathogens, facilitating enhanced accumulation in lymphoid tissues such as lymph nodes and promoting efficient uptake by APCs. This targeting capability allows for the simultaneous delivery of antigens and immune activators to specific immune cells [130, 131]. In this context, TAA-specific T cells can be directly activated, and TAAs may engage T cells through cross-presentation by APCs, resulting in a robust immune memory that effectively inhibits tumor initiation and growth, including drug-resistant tumor stem cells [78, 132].

Recent advancements by Wu et al. developed a nanoparticle-based cancer vaccine that that not only significantly inhibits tumor growth in vivo but also converts primary tumors into extensive antigenic libraries. This breakthrough supports the development of highly personalized cancer vaccination approaches [78]. Another innovation, the dClip-LNP/siRNA nano-vaccine, also exhibited promising anti-tumor effects, substantially reducing TIM3 expression in DCs and enhancing antigen presentation, which subsequently activated T cells [79]. Moreover, a novel nanocomposite, the CS-TEMPO-OVA vaccine, effectively suppressed the growth of melanoma by inducing immune response, activating cytotoxic T cells, and promoting macrophage M1 polarization. When conjunction with aPD-1, it significantly enhanced CTL infiltration and elicited a potent systemic anti-tumor response that effectively curtails tumor metastasis [80].

Despite these technological advancements, the performance of cancer nano-vaccines in clinical settings has been mixed, potentially due to functional deficiencies in antigen-loaded DCs [133]. Additionally, the complex manufacturing processes and high costs associated with these vaccines, along with their limited antigen expression, highlight the need for integrating them with other therapeutic modalities in clinical applications [134, 135].

## Adjuvants and their mechanisms in cancer vaccines

**Fig. 2:**
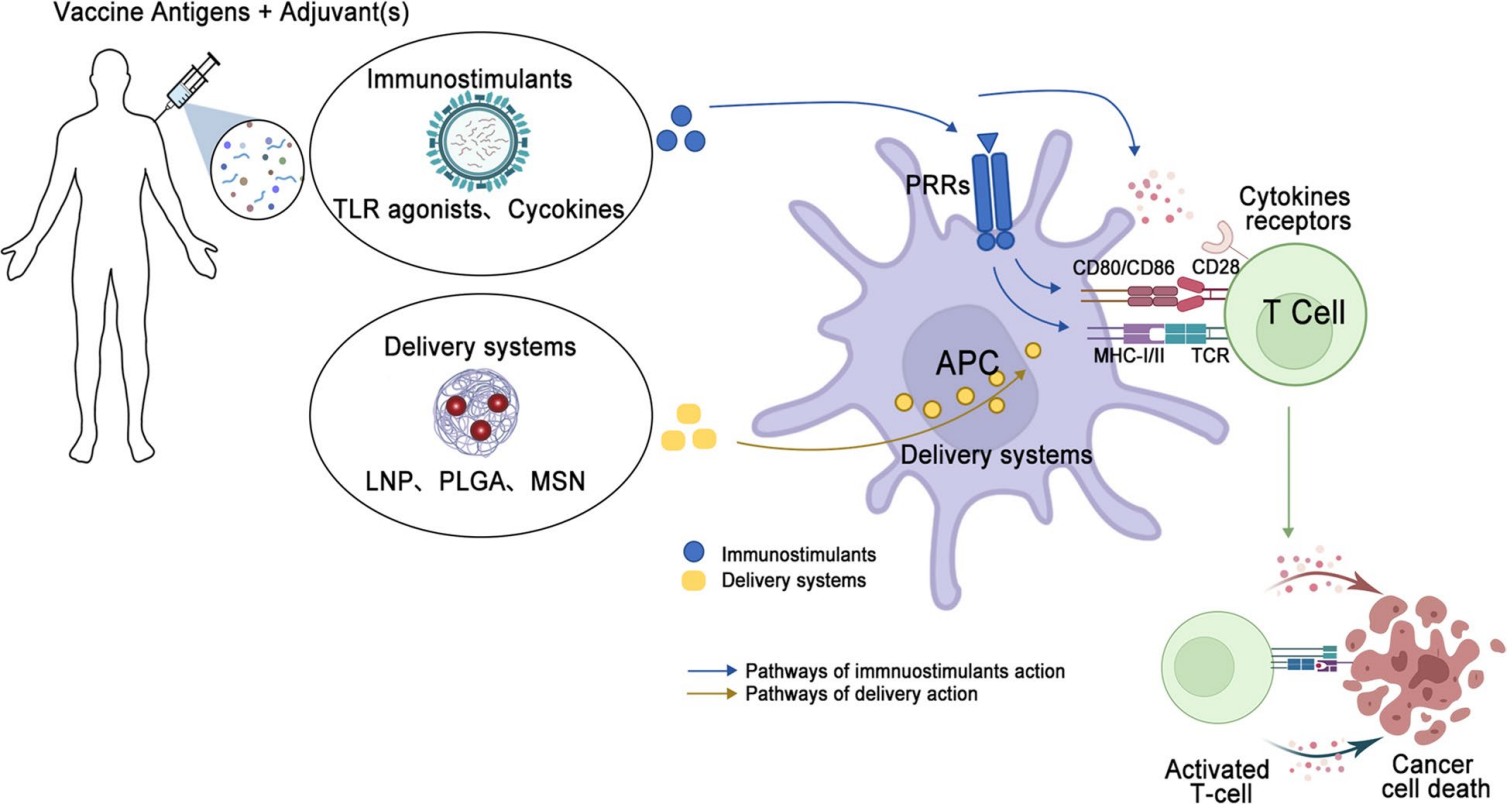
General schematic of the mechanism of action of adjuvanted cancer vaccines. The classification of adjuvants can be broadly divided into two categories: immunostimulants and delivery systems. Immunostimulants, such as TLR. agonists, have the capacity to activate pattern-recognition receptors (PRRs) on antigen-presenting cells (APCs), thereby enhancing the presentation of antigens via major histocompatibility complex (MHC) molecules. Furthermore, the activation of PRRs results in the upregulation of cytokine and co-stimulatory molecule expression, thereby enhancing the co-stimulatory signals CD80/CD86. Conversely, cytokines can directly promote the proliferation and activation of tumor-specific T cells. Delivery systems, including LNPs, PLGA, and MSN, facilitate antigen presentation on MHC molecules. The interaction between APCs and T cells results in the generation of greater numbers and types of multifunctional T cells, which ultimately lead to cancer cell death. TLR, toll-like receptor; LNP, lipid nanoparticles; MSN, mesoporous silica nanoparticles; PLGA, poly(lactide-co-glycolide); APC, antigen presenting cells

As previously noted, DCs activation is paramount in the efficacy of cancer vaccines. Recent advancements in vaccine platforms have introduced delivery systems with improved immunogenicity through enhanced physical properties. Nevertheless, the prevailing immunosuppressive milieu within tumors necessitates the inclusion of adjuvants to boost the efficacy of the vaccine platform and stimulate the immune system [136]. Cancer vaccine adjuvants, immunogenic substances that boost the adaptive immune response, thereby increasing the efficacy and persistence of responses to tumor antigens. This process notably enhances the activation of T and B cells, boosting the immune system’s tumor-fighting capabilities [137]. Moreover, adjuvants improve the efficiency with which APCs, like DCs, recognize and present these antigens, facilitating a more focused immune response [138]. Furthermore, they extend the immune response’s duration, improve immunological memory, and diminish the risk of tumor recurrence [139]. Generally, cancer vaccine adjuvants are broadly categorized as delivery systems or immunostimulants [140] (Fig. 2). Delivery systems facilitate the uptake of antigens by APCs via endocytosis, though this can sometimes lead to suboptimal T cell activation. In contrast, immunostimulants, derived from TAAs with low immunogenicity, are designed to enhance immune activation [141–143]. Effective adjuvants ideally improve antigen presentation, stimulate robust immunity, and counteract tumor-induced immunosuppression.A review of various cancer vaccine adjuvants is summarized in Table 2.

**Table 2:** Summarize of cancer vaccine adjuvants

Type	Classification	Related Cancer Vaccines	Ref
TLR agonists	TIL3	NEO-PV-01	[18]
		GEN-009	[144]
		DC	[145]
	TIL4	MAGE-A3 protein vaccine	[146]
		Cervarix	[147]
	TIL7/8	GTL001	[148]
		mRNA vaccine	[149]
	TIL9	MAGE-A3 protein vaccine	[150]
Cytokines	IL-2	Melan-A	[151]
		IL-2 DC	[152]
		TG4010	[153]
	GM-CSF	GVAX	[65]
		Tyrosinase peptide containing GM-CSF	[154]
		E75	[155]
		Sipuleucel-T	[67]
		GP2	[89]
		Galinpepimut-S	[156]
	IFN-α、IFN-γ	IFN-α peptide vaccine	[157]
		Autologous vaccine + IFN-α/IFN-γ	[158]
	IL-12	Fusion of DC cells	[159]
Lipid-based adjuvants	Cationic Liposomes	BNT111/112/113/115/116	[64, 160]
	LNP	IVAC MUTANOME	[161]
		BNT122	[64]
		mRNA-5671	[160]
		mRNA-4157	[59]
Adjuvant for pellets	Silicon	uGCVax	[162]
	MSN	DC	[163]
	PLGA	PRECIOUS-01	[164]
	VLP	Cervarix	[165]
		Gardasil	[166]
	Aluminium salt	Aluminium hydroxide	[167]
Emulsion adjuvants	Montanide ISA-51	Cimavax-EGF	[168]
		Melanoma-associated helper peptides	[141, 169]
		RV001	[170]

*TLR* toll-like receptor, *DC* dendritic cell, *GM-CSF* granulocyte macrophage colony-stimulating factor, *MSN* mesoporous silica nanoparticles, *PLGA* poly(lactide-co-glycolide), *VLP* Virus-like particles, LNP Lipid nanoparticles

Among immunostimulants, Toll-like receptor (TLR) agonists, such as TLR3, TLR4, TLR7/8, and TLR9, are pivotal for enhancing antigen presentation and co-stimulatory signaling. Notably, Synthetic double-stranded RNA agonists like Poly-I:C and its derivative Poly-ICLC (TLR3 ligands) stimulate IFN-β production and pro-inflammatory cytokines such as IL-6 and IL-12, facilitating antigen cross-presentation to CD8 + T cells and promoting Th1-skewed immunity [171–173]. The neoantigen cancer vaccine NEO-PV-01, which employs Poly-ICLC as an adjuvant, showed tolerability in advanced solid tumor patients during a phase Ib trial. Subsequent analyses indicated that the vaccine-induced T cells displayed a cytotoxic phenotype, capable of migrating to tumors and inducing cell death [52]. Similarly, 3-deacyl-monophosphoryl lipid A (MPL), a TLR4 activator derived from lipopolysaccharides (LPS), enhances Th1 responses via NF-κB activation and cytokine release [174]. AS04, an adjuvant combining MPL with aluminum salts, is utilized in the HPV vaccine Cervarix to boost antibody production and activate antigen-specific T cells [147]. Cytokines such as IL-12 and IL-2 are promising cancer vaccine immunostimulants due to their ability to enhance tumor-specific T cell proliferation and activation, promoting cytotoxic responses and Th1 polarization [152, 159]. Other cytokines, such as IFN-α, has also shown potential as an adjuvant [157, 175]. However, cytokine therapies carry risks of adverse effects like nausea, vomiting, fever, chills, fatigue, and headaches, often linked to dosage, necessitating cautious clinical use [176].

Liposomes, another adjuvant class, improve antigen delivery, stability, and immune activation [177]. Cationic liposomes, with high affinity for negatively charged cancer cell membranes, facilitate more efficient uptake by tumor cells and protect biomolecules like RNA and DNA from enzymatic degradation, facilitating endocytosis by APCs [178, 179]. Numerous studies have assessed the anti-tumor efficacy of cationic liposome-mRNA formulations, including BNT111, BNT112, BNT113, BNT115, and BNT116 [64, 160]. Lipid nanoparticles (LNPs), composed of ionizable lipids, phospholipids, cholesterol, and polyethylene glycol, are optimized for mRNA cancer vaccines delivery. Ionizable lipids bind RNA, while phospholipids and cholesterol enhance bilayer stability and fluidity. Clinical trials are exploring LNP-based cancer vaccines like autogene cevumeran for pancreatic ductal adenocarcinoma and LNP-mRNA-5671 targeting KRAS mutations in NSCLC [160, 180].

Particulate adjuvants are extensively used in cancer vaccines to enhance antigen delivery, boost immune activation, and trigger inflammatory responses [162–166]. Among these, mesoporous silica nanoparticles (MSNs) are notable for their high capacity and easy modification [167]. Their application in cancer vaccines has shown promise by increasing cytotoxic T lymphocyte (CTL) production and reducing tumor progression in *vivo* [163]. Virus-like particles (VLPs), derived from viral capsid proteins, possess a structured design that facilitates strong B cell activation by cross-linking B cell receptors, even without helper T cells. VLP-based vaccines, such as Cervarix and Gardasil for HPV, are widely utilized [165]. Oil-in-water emulsions, particularly Montanide ISA-51, act as effective adjuvants by stabilizing antigens and enhancing immune responses [168]. Compared to Freund’s adjuvant, often used in veterinary medicine, Montanide ISA-51 offers superior safety and stability [141]. It is currently approved for lung cancer vaccines and, when combined with peptide vaccines, enhances CD8 + and CD4 + T cell infiltration, as observed in melanoma studies [169, 181, 182].

## Cancer vaccines combined with other treatments

**Fig. 3:**
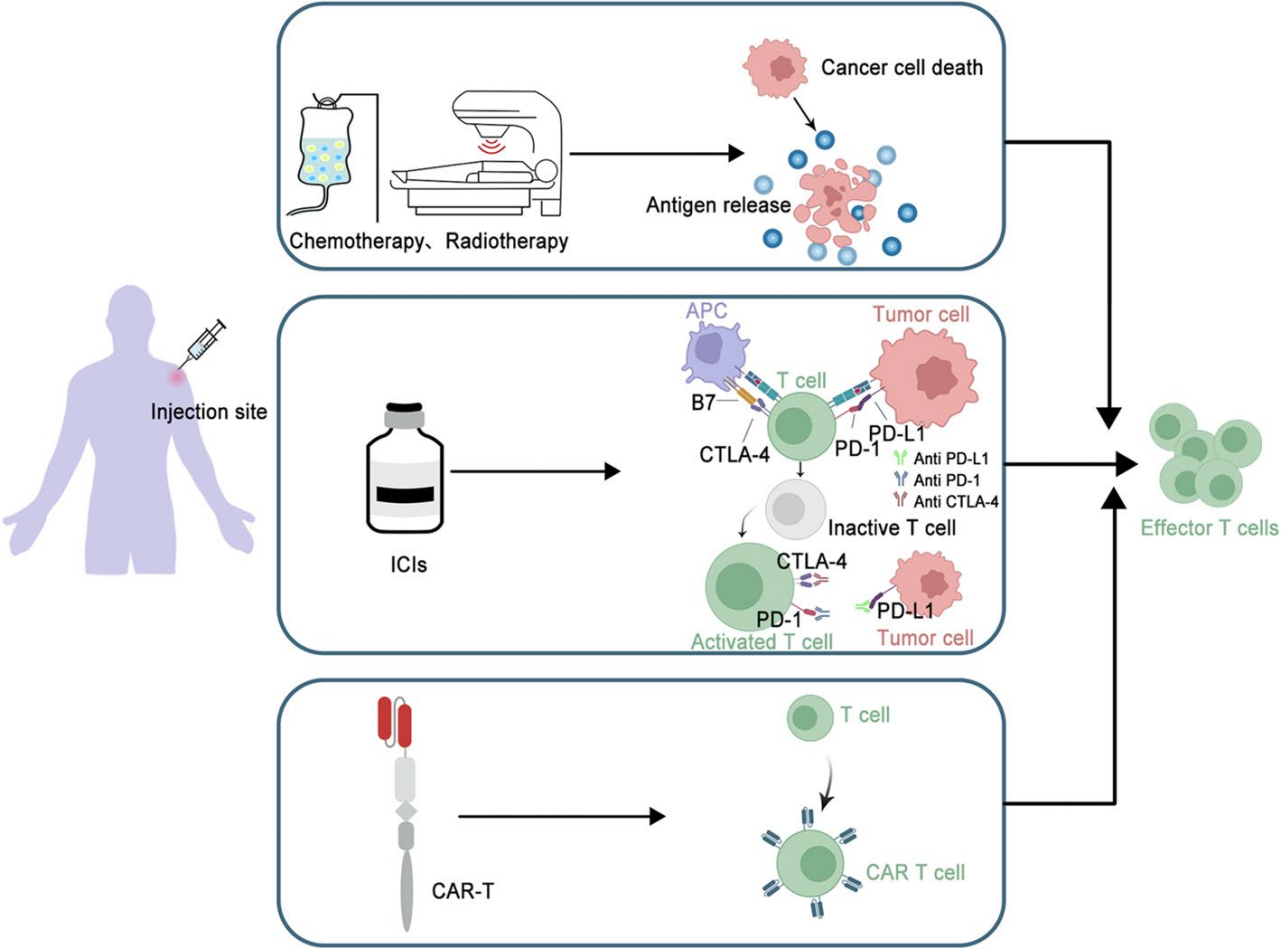
Diagram of the mechanism of different combinations of therapies after a patient has been vaccinated against cancer. Conventional therapies, such as chemotherapy and radiotherapy, complement this process by directly affecting tumor cells or indirectly eliminating immunosuppressive cells. Immune checkpoint inhibitors (ICIs) have the ability to unlock immunosuppression and stimulate T-cell activation by blocking signaling pathways on the surfaces of T-cells and cancer cells. Chimeric antigen receptor (CAR) T cells therapy transforms T cells, which are ordinary "fighters," into "super fighters" by attaching the localization navigator CAR. The activation of effector T-cells and improvement in the clinical efficacy of neoantigenic cancer vaccines can be achieved through the use of different therapeutic combinations. ICIs, immune checkpoint inhibitors; PD-1, programmed cell death 1; PD-L1, programmed cell death ligand 1; CTLA-4, cytotoxic T-lymphocyte antigen 4; CAR-T, chimeric antigen receptor T

The restricted expression of tumor antigens, coupled with the heterogeneity of tumors, diminishes the clinical effectiveness of cancer vaccines as monotherapy, especially in patients who have received multiple lines of treatment. Consequently, combination therapies are poised to dominate future cancer treatment strategies (Table 3 & Fig. 3).

**Table 3:** Summary of some vaccine combination therapies in phase II/III clinical trials with positive results

Vaccine	Combination regimens	Phases (Trial ID)	Cancer type	Ref
CervISA	+ carboplatin/paclitaxel	II (NCT02128126)	Cervical cancer	[183]
DCVAC/LuCa	+ carboplatin/paclitaxel	II (NCT02107937)	Ovarian cancer	[184]
SurVaxM	+ temozolomide	II (NCT02455557)	GBM	[185]
Avipox vaccine	+ EBRT	II (NCT00005916)	Prostate cancer	[186]
mRNA4157	+ Pembrolizumab	IIb (KEYNOTE-942)	NSCLC, Melanoma	[105, 187]
GVAX	+ Nivolumab + Urelumab	II (NCT02451982)	Pancreatic cancer	[65]
EO2401	+ Bevacizumab	Ib/IIa (NCT04116658)	Glioblastoma	[187]
IO102/IO103	+ Pembrolizumab	II (NCT05077709)	NSCLC	[188]
GRANITE	+ Ipilimumab + Atezolizumab + 5-FU + Bevacizumab	II/III (NCT05141721)	CRC	[189]
GNOS-PV02	+ Pembrolizumab	Ib/IIa (NCT04251117)	HCC	[190]
T-VEC	+ Pembrolizumab	III (MASTERKEY-265)	Melanoma	[191]
ISA101	+ Nivolumab	II (NCT02426892)	Solid tumors	[192]
BNT211	+ CARVac	I/IIa (NCT04503278)	Solid tumors	[193]

*EBRT* External beam radiotherapy, *GBM* glioblastoma, *HNSCC* head and neck squamous cell carcinoma, *NSCLC* non-small-cell lung cancer, *CRC* colorectal cancer, *HCC* hepatocellular carcinoma

### Cancer vaccines combined with chemotherapy

Tumors often promote the differentiation or activation of immunosuppressive cell subpopulations to evade immune detection. Combining cancer vaccines with chemotherapy not only enhances efficacy but also mitigates the risk of immune escape resulting from antigen loss [184, 194, 195]. The destruction of the tumor and its surrounding tissues by chemotherapy leads to the release of additional antigens, which facilitates T cell recruitment to the TME. This is especially beneficial in tumors characterized by low mutational burdens and immunologically ‘cold’ environments [183, 196]. Vaccines can also effectively target existing malignant cells by triggering polyclonal T cell response, particularly through neoantigens derived from tumor-specific mutations that bypass central tolerance, thereby generating robust immune response [197, 198].

Numerous clinical trials are investigating the synergistic effect of chemotherapy in enhancing vaccine-induced immune response. For instance, a phase II study of the HPV vaccine CervISA demonstrated that chemotherapy in advanced cervical cancer patients eliminated suppressive myeloid cells without depleting lymphocytes, resulting in a robust T-cell response to a synthetic long peptide vaccine targeting anti-HPV16 E6/E7 [183]. Additionally, a study examining the efficacy and safety of the DC vaccine DCVAC/LuCa in combination with chemotherapy for driver-negative advanced NSCLC revealed one-year and two-year OS rates of 72.73% and 52.57%, respectively, with a median PFS of 8.0 months and a favorable safety profile [184]. Furthermore, a phase IIa trial combining SurVaxM, a synthetic peptide vaccine targeting survivin, with TMZ as first-line treatment for GBM demonstrated significant improvement in median PFS and OS, reaching 11.4 months and 25.9 months, respectively [185].

### Cancer vaccines combined with radiotherapy

Immune response induced by cancer vaccines can be amplified when combined with radiotherapy. Huang KCY, et al. found that murine splenocytes treated with a neoantigen-augmented iPSC cancer vaccine and radiotherapy secreted more IFN-γ and exhibit enhanced cytotoxicity against cancer cells [199]. Radiotherapy can stimulate anti-tumor immune response through multiple mechanisms, such as expanding the T cells, attracting T cells to the radiation site, and rendering irradiated cells more susceptible to T cell-mediated destruction [200]. Moreover, radiotherapy not only targets local tumor cells but also enhances immune response in distant lesions through systemic effects.

Current clinical application of cancer vaccines combined with radiotherapy have shown promising results. A study by Gulley et al. indicated that an avipox virus vaccine encoding PSA exhibited favorable anti-tumor effects when administered alongside radiotherapy. Of the 17 patients in the combination group, 13 exhibited at least a three-fold increase in PSA-specific T cells, whereas no increase was detected in the radiotherapy alone group [186]. In triple-negative breast cancer, radiotherapy was found to enhance the cellular expression of MHC-I/II molecules and promote specific neoantigen presentation both in vitro* and *in vivo [201]. Nonetheless, radiotherapy could also produce immunosuppressive effect that may diminish the adaptive and innate immune response [202, 203]. Therefore, close monitoring of the patient's immune status is essential, and treatment regimens should be adjusted accordingly.

### Cancer vaccines combined with ICIs

Neoantigen vaccines are capable of generating antigen-specific T cells and promoting their differentiation into effector T cells, exhibiting strong tumor-specific immunogenicity. However, these T cells often express immunosuppressive markers. Blocking the PD-1/PD-L1 signaling pathway could reverse this immunosuppressive microenvironment [204, 205]. As a result, the combination of cancer vaccines with ICIs may yield synergistic anti-tumor effect. For example, the KEYNOTE-942 trial indicated that the mRNA-4157 vaccine in combination with pembrolizumab achieved a 50% overall remission rate and a median PFS of 9.8 months in patients with HPV-negative head and neck squamous cell carcinoma (HNSCC), surpassing previous outcomes associated with pembrolizumab monotherapy [89]. Crucially, recent data from this trial indicate that the combination of mRNA-4157 with pembrolizumab is associated with a statistically significant and clinically meaningful enhancement in RFS compared to pembrolizumab monotherapy, demonstrating a 44% reduction in the risk of disease progression or mortality among patients with resected high-risk stage IIIB/C/D and IV melanoma [187]. Consequently, the mRNA-4157/V940 regimen in conjunction with pembrolizumab received Breakthrough Therapy and PRIME (Priority Medicines) designations from the FDA for the adjuvant treatment of high-risk stage III/IV melanoma [136]. However, the trial faced suspension due to adverse events related to the combination therapy and instances of disease recurrence following pembrolizumab monotherapy.

EO2401, a cancer vaccine that significantly expands effector memory CD8 + T cells, showed promising results in combination with nivolumab, with or without bevacizumab, prolonging treatment duration and improving ORR, DCR, and PFS in patients with recurrent GBM [206]. Furthermore, preliminary data from a clinical trial indicated that the combination of IO102/IO103 with pembrolizumab exhibited encouraging early clinical activity in lung adenocarcinoma patients with high PD-L1 expression, with 53.3% of patients demonstrating substantial tumor shrinkage and 26.7% achieving stable disease [188]. Results from the phase II trial (NCT05141721) in first-line metastatic microsatellite stable colorectal cancer (MSS-CRC) demonstrated that treatment with GRANITE (GRT-C901/GRT-R902), a neoantigen-based mRNA vaccine, yielded an 18% reduction in the risk of disease progression or death across all patients compared to the control group, with a more pronounced 48% reduction in high-risk patients (> 90% with liver metastases). The median PFS was 11.57 months in the GRANITE cohort versus 7.06 months in the control arm [189]. These findings suggest that the GRANITE cancer vaccine may effectively activate a patient's immune system to combat this “cold” tumor type, which traditionally exhibits lower responsiveness to immunotherapy. Additionally, in the multicenter GT-30 study, the personalized cancer vaccine GNOS-PV02, when combined with a PD-1 inhibitor, was administered to 36 patients with advanced HCC. Of these, three patients achieved complete tumor disappearance, while an additional eight patients attained a partial response (PR), resulting in an overall objective response rate (ORR) of 30.6%. In contrast, the ORR in the KN-240 study of pembrolizumab monotherapy for advanced HCC was only 17.0%, underscoring the significant enhancement of immunotherapeutic efficacy when supplemented with the personalized vaccine [190].

Nevertheless, this approach encounters several challenges, including high costs, side effects and inconsistent efficacy [207, 208]. As mentioned earlier, mRNA-4157 did not demonstrate efficacy in a phase I trial when administered alongside pembrolizumab for CRC. Future research must investigate strategies to overcome these obstacles for broader clinical applicability [60].

### Cancer vaccines combined with CAR-T therapies

CAR-T therapy represents an innovative form of precision-targeted immunotherapy. Nevertheless, the administration of a single high-dose infusion can lead to transient cytokine overproduction, resulting in CRS that significantly limits its clinical practice [209]. When combined with cancer vaccines, it may be possible to reduce the quantity of CAR-T cells infused and establish long-term memory against specific tumor antigens [210].

Furthermore, Ma et al. developed a vaccine that targets the same antigens recognized by CAR-T cells, utilizing a mechanism distinct from that of traditional tumor antigens, thereby augmenting the capability of CAR-T cells to effectively target solid tumors and significantly enhancing the overall efficacy of CAR-T therapy [211]. Their preclinical investigations demonstrated that the vaccine not only activated CAR-T cells but also stimulated other T cell populations capable of recognizing additional tumor antigens, thus addressing previous challenges associated with incomplete tumor eradication by CAR-T therapies [14]. Emerging evidence further indicates that mRNA encoding CAR-T cell-targeted antigens can selectively stimulate APCs in lymph nodes to express these antigens, promoting CAR-T cell expansion in patients and showing promising efficacy in solid tumors [193, 212]. For instance, the trail (NCT04503278) evaluated the efficacy of an RNA vaccine in conjunction with CAR-T cells targeting the tumor neoantigen claudin 6 in solid tumors. Preliminary results from 21 evaluable patients revealed an optimal ORR of 33% and a DCR of 67%, including one patient achieving CR, alongside manageable side effects, highlighting the potential of cancer vaccines to enhance the durability of CAR-T cell responses [193]. This synergistic effect may broaden clinical applicabilities and enhance durable clinical remissions. Nevertheless, significant challenges persist, such as high costs, intricate manufacturing processes, and potential safety risk.

## Clinical challenge

Translating cancer vaccines into clinical practice involves multiple stages, including vaccine preparation, preclinical investigation, management of AEs, and refined combination therapies.

### Cancer vaccine preparations

First, it is essential to identify the antigens that can elicit an immune response. Vaccines targeting tumor-derived neoantigens should ideally induce a more robust immune response with reduced autoimmune-related toxicity [213]. Consequently, neoantigens are identified through tumor genomic DNA sequencing to create personalized recombinant vaccines. This process may employ genetic engineering techniques to produce the antigens or utilize viral vectors for targeted delivery [50]. Furthermore, immunosuppressive cells and factors in the TME can hinder the anti-tumor response [214]. Therefore, developing cancer vaccines necessitates strategies to eliminate these immunosuppressive elements.

### Selection of animal models

The application of cancer vaccines requires effective preclinical studies, wherein preliminary safety and efficacy are evaluated. The selection of appropriate models is crucial in this context [215]. Suitable animal models should fulfill several main criteria: 1) preservation of immune cell subtypes; 2) HLA-matching between tumor and immune system; and 3) alignment of the immune response pathway with that of humans. These conditions ensure that animal models can accurately reflect human immune response and tumor characteristics.

Commonly utilized mouse models include the human peripheral blood mononuclear cell (Hu-PBMC) and human hematopoietic stem cell (Hu-HSC) models. The Hu-PBMC model can rapidly reconstitute mature, activated human T lymphocytes. However, the risk of lethal graft-versus-host disease (GvHD) limits its use [216, 217]. Conversely, the Hu-HSC model reconstitutes various human hematopoietic cells, including T, B, natural killer (NK), and myeloid cells. However, the lack of a human thymus leads to suboptimal T cell development and inefficient antibody class switching [218]. Consequently, the Hu-PBMC model is more suitable for early drug efficacy studies focused on T or NK cells, while the Hu-HSC model is better for evaluating long-term memory effects or sustained anti-tumor responses. Additionally, integrating cell line-derived xenografts (CDX) or patient-derived xenografts (PDX) with humanized mice created from Hu-PBMC or Hu-HSC, along with precise sequencing, can provide a more comprehensive immune system for assessing the efficacy of cancer vaccines [219].

Despite the critical role of animal testing in drug development and toxicity assessment, the FDA approved the first new drug-derived entirely from preclinical data generated using organ-on-a-chip technology-in August 2022 (NCT04658472) [220]. This landmark decision signifies a pivotal shift, as organ-on-a-chip experiments have, for the first time, supplanted traditional animal studies in certain contexts. Subsequently, the FDA announced the removal of the general requirement for animal testing prior to human clinical trials, marking a significant transformation in drug safety regulations. Emerging alternative non-animal technologies, such as organ microarrays and organoids, may herald key trends in the selection of cancer vaccine models [221]. Nonetheless, researchers must also consider how to ensure the safety of participants in early-phase clinical trials.

### Management of adverse events (AEs)

The AEs associated with cancer vaccines are multifaceted, influenced by targeted antigens, various dosage forms, different adjuvants, and the potential for autoimmunity induced by immunostimulants. Generally, cancer vaccines are linked to mild AEs, such as injection site reactions [222]. Patients may experience infusion reactions characterized by flu-like symptoms and myalgia, typically mild to moderate in severity and resolving within 24–48 h following administration [67]. Moreover, certain studies indicated a heightened incidence of cerebrovascular events in the sipuleucel-T vaccine cohort compared to placebo [223, 224]. Rare AEs include hypoxia, tachypnea, hypoxemia, wheezing, bronchospasm, and cardiac arrhythmias [225]. Serious AEs, including acute kidney injury, B-cell lymphoma, and lymphadenopathy, could also occur [226].

The AEs associated with tumor antigens are generally low, as TAAs/TSAs are expressed at higher levels in cancer cells compared to normal cells [227]. Additionally, reports indicated that small molecule adjuvants with TLR agonists may induce AEs, including headache, fever, and cytokine storms [228, 229]. It's worth noting that the combination of cancer vaccines with ICIs does not seem to enhance the incidence of AEs [230, 231].

In summary, although cancer vaccines demonstrate significant potential, the management of safety and adverse events (AEs) necessitates careful monitoring and evaluation. Current research on innovative mRNA cancer vaccines seeks to improve efficacy against specific cancers while minimizing AEs.

## Conclusions

As the field of immuno-oncology has developed and our comprehension of the mechanisms of immune evasion from tumors has advanced, ICIs and ACT therapy have demonstrated the feasibility of immunotherapy in both solid and hematological tumors. Antigens can be utilized in a multitude of platforms, including peptides, nucleic acids, viruses, cellular components, DCs, and even nano-vaccines. mRNA vaccines have demonstrated considerable advancement in clinical trials due to their efficacy and adaptability, and the successful completion of preclinical and clinical studies may influence the trajectory of future therapeutic platform design.

Extensive research underscores the efficacy of cancer vaccines, with a focus on enhancing precision and versatility. A combination of different vaccine platforms, appropriate tumor antigens, immunostimulants, and promising combination therapy strategies is necessary to overcome tumor resistance. Ongoing efforts are essential to optimize vaccine design, improve delivery systems, and refine combination therapies.

Future research should prioritize several key areas. First, the screening for individualized antigens and the development of personalized cancer vaccines are recommended, facilitated by advancements in NGS and continuously optimized algorithms that enhance the prediction of neoantigens. Second, it is crucial to develop optimal vectors for vaccines and refine adjuvants to improve anti-tumor efficacy. Additionally, addressing the immunosuppressive TME is vital. Strategies may include inhibiting immunosuppressive cytokines (e.g., TGF-β), delivering immunomodulatory cytokines (e.g., GM-CSF), and targeting angiogenesis to enhance the accumulation of tumor-infiltrating lymphocytes.

## Data Availability

Not applicable.

## Abbreviations

ICIs: Immune checkpoint inhibitors
CTLA-4: Cytotoxic T-lymphocyte antigen 4
CAR-T: Chimeric antigen receptor T
TME: Tumor microenvironment
TAAs: Tumor-associated antigens
TSAs: Tumor-specific antigens
LAG3: Lymphocyte activating 3
PD-1: Programmed cell death 1
CTLs: Cytotoxic T lymphocytes
Th1: Type 1 T helper
GM-CSF: Granulocyte macrophage-colony stimulating factor
PD-L1: Programmed cell death ligand 1
IFN-γ: Interferon gamma
DC: Dendritic cells
NSCLC: Non-small cell lung cancer
DMFS: Distant metastasis-free survival
HLAs: Human leukocyte antigens
APC: Antigen presenting cell
MHC-I: Histocompatibility complex class I
RCC: Renal cell carcinoma
RFS: Recurrence-free survival
DFS: Disease-free survival
AEs: Adverse events
hTERT: Human telomerase reverse transcriptase
WT1: Wilms tumor-1 protein
PSMA: Prostate-specific membrane antigen
IL-12: Interleukin-12
GBMs: Glioblastomas
PSA: Prostate-specific antigen
ORR: Overall response rate
DCR: Disease control rate
mCRPC: Metastatic castration-resistant prostate cancer
PAP: Prostatic acid phosphatase
nGBM: Newly diagnosed gliomas
rGBM: Recurrent gliomas
T-VEC: Talimogene laherparepvec
DRR: Durable remission rate
CEA: Carcinoembryonic antigen
AFP: Alpha fetoprotein
CA125: Cancer antigen 125
MUC1: Mucin 1
CR: Complete response
NY-ESO-1: New York esophageal squamous cell carcinoma 1
MAGE-A3: MAGE family member A3
TPTE: Transmembrane phosphatase with tensin homology
EGFR: Epidermal growth factor receptor
TMZ: Temozolomide
EBV: Epstein-Barrvirus
HPV: Human papillomavirus
HTLV-1: Human T-lymphotropic virus type 1
TLRs: Toll-like receptors
MPLA: Monophosphoryl lipid A
MSN: Mesoporous silica nanoparticles
VLPs: Virus-like particles
BCRs: B cell receptors
HNSCC: Head and neck squamous cell carcinoma
TGF: Transforming growth factor
VEGF: Vascular endothelial growth factor
